# Packed Red Blood Cell and Whole Blood Perfusates during *Ex Vivo* Normothermic Perfusion for Assessment of High-Risk Donor Kidneys

**DOI:** 10.34067/KID.0000000815

**Published:** 2025-05-07

**Authors:** Armin Ahmadi, Heiko Yang, Kuang-Yu Jen, Sili Fan, Ivonne Palma, Junichiro Sageshima, Naeem Goussous, Baback Roshanravan, Richard V. Perez

**Affiliations:** 1 Division of Nephrology, Department of Medicine, University of California, Davis, California; 2 Department of Urology, University of California San Francisco, San Francisco, California; 3 Department of Pathology and Laboratory Medicine, University of California at Davis, Sacramento, California; 4 Department of Biostatistics, School of Medicine, University of California, Davis, California; 5 Division of Transplant, Department of Surgery, University of California Davis Health, Sacramento, California

**Keywords:** kidney biopsy, metabolism, transplantation, biomarkers, metabolomics

## Abstract

**Key Points:**

- Compared with whole blood (WB) kidneys perfused with a packed red blood cells perfusate exhibited superior kidney functional parameters.
- Metabolomics assessment showed drastic metabolic aberration in the WB group involving amino acid, carbohydrate, and mitochondrial energy metabolism.
- Tissue lipid profiling demonstrated that WB was associated with accumulation of tissue membrane/structure components including glycerolipids and ceramides.

**Background:**

*Ex vivo* normothermic perfusion (EVNP) with a blood-based perfusate has the potential to both assess viability of and repair high-risk organs before transplantation. The optimal perfusate is yet to be established.

**Methods:**

We assessed hemodynamic, functional, and metabolic changes of eight paired high-risk human kidneys perfused with either a leukocyte-depleted packed red blood cell (PRBC) or a whole blood (WB) perfusate during a 3-hour EVNP.

**Results:**

After a mean cold ischemia time of 54 hours, all kidneys showed high renal blood flow through perfusion. Renal resistance increased for both groups during the first hour and then decreased to similar terminal values. The kidneys perfused with PRBC had 55 ml/min greater renal blood flow (95% confidence interval, 21 to 89; *P* = 0.004) and higher total urine output (145 versus 25 ml, *P* = 0.002) compared with the WB group. Urinary acute kidney biomarkers of neutrophil gelatinase-associated lipocalin and kidney injury molecule-1 were also significantly lower (mean differences of 281 and 2.1 ng/ml, respectively; *P* < 0.01) in the PRBC perfused kidneys. Compared with PRBC, within-group tissue metabolic profiling revealed a similar (23% versus 18%) but a more pronounced alteration involving (branched chain) amino acid and mitochondrial energy metabolism in the WB group. Similarly, lipid profile temporal changes showed that WB groups were highlighted by elevation of plasma membrane and structure lipids including glycerolipids, sphingolipids, and steroids. The PRBC group had minimal temporal tissue lipid profile changes.

**Conclusions:**

Compared with WB, PRBC perfusion is superior in mitigating postischemia damage and facilitating function and metabolic recovery of high-risk kidneys subjected to long cold ischemia times during a 3-hour EVNP.

## Visual Abstract

**Figure:**
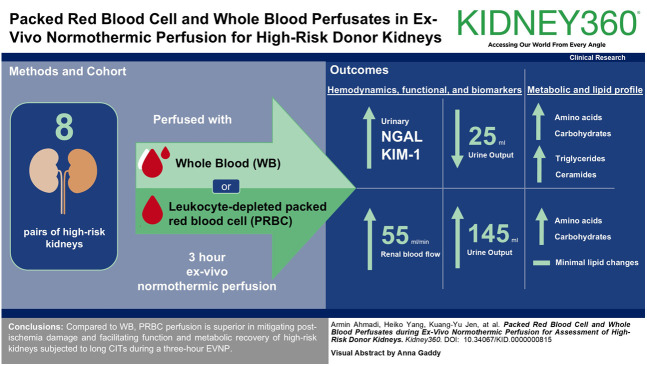

## Introduction

The most significant obstacle in kidney transplantation is the shortage of available donor organs.^1^ Generally, the suitability of kidneys for transplant is determined based on a variety of donor factors including donor age and comorbidities, duration of cold ischemic time, renal dysfunction, and appropriateness for the intended recipient.^2^ To address the shortage, the donor pool has been expanded to include higher risk organs such as those with AKI, donation after circulatory death, and older donors with defined comorbidities previously termed extended criteria donors. Although improved survival has been demonstrated after transplantation with higher-risk organs,^3^ many kidneys procured with the intention to transplant are discarded.^4^

Over the past two decades, there has been a growing emphasis on exploring dynamic preservation strategies, including hypothermic machine perfusion (HMP) and normothermic machine perfusion, to improve organ viability and transplant outcomes.^5–7^ HMP is widely used in deceased donor kidney transplantation to preserve the organ during transport and mitigate cold ischemic injury.^8,9^ By circulating a cold perfusate solution through the graft for several hours postretrieval, HMP has been shown to reduce delayed graft function (DGF) and improve 3-year graft survival compared with static cold storage.^10,11^

*Ex vivo* normothermic perfusion (EVNP) is another emerging technology that has successfully preserved marginal donor organs, including the heart, lung, liver, and kidney.^12–15^ EVNP has been reported as a means of preserving and assessing the viability of high-risk kidneys by restoring metabolic physiologic conditions before transplantation.^15,16^ Compared with conventional static cold storage, EVNP enhances organ viability by reducing ischemia-reperfusion injury and DGF, ultimately improving transplantation outcomes. With re-establishment of normal metabolism using a blood based perfusate, EVNP can replenish mitochondrial energy stores with increased ATP levels^17^ and improve renal allograft function after ischemia induced injury.^15,18,19^ Most current kidney perfusion protocols involve continuous perfusion with red blood cells or an oxygen carrier alternative and glucose and amino acids as energy sources. However, the optimal perfusate for use during EVNP to efficiently improve viability, function, and metabolic health is yet to be determined.

A packed red blood cell (PRBC)–based perfusate has been used in most studies due to evidence, suggesting that leukocytes and platelets contribute to ischemia reperfusion injury.^20–23^ On the other hand, whole blood (WB) has been shown to be superior to a PRBC perfusate in an experimental model of heart transplantation, presumably because of its more accurate reproduction of normal physiologic conditions.^24^ Here, we performed a 3-hour EVNP, during which we assessed various kidney hemodynamics and functional parameters, and we also performed untargeted metabolomics and lipidomics using kidney cortical tissues from eight paired kidneys. The purpose of this study was two-fold: first, to compare *ex vivo* renal physiology and function using the PRBC perfused EVNP used clinically compared with a WB EVNP system which simulates normal physiologic conditions, and second, to characterize their respective metabolic implications during a 3-hour EVNP period. In addition, by using tissue lipid profile assessment, we aimed to compare the severity of reperfusion injury and inflammation on tissue/cell damage during perfusion and to develop lipid-based biomarkers of necrosis and tissue injury.

## Methods

### Deceased Donor Kidneys

**Figure 1:**
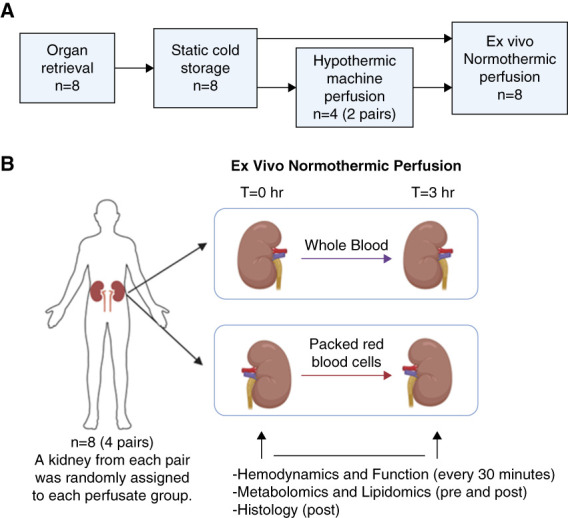
**Experimental design of the study.** (A) Kidney assessment timeline and (B) EVNP experimental design (*n*=8). All paired underwent static cold storage, and two pairs (*n*=4) underwent hypothermic machine perfusion before EVNP. One kidney from each pair was randomly assigned to whole blood or packed red blood cell perfusion. EVNP, *ex vivo* normothermic perfusion.

The study was reviewed by and considered exempt from the Institutional Review Board at our institution and approved by the Human Anatomical Specimen and Tissue Oversight Committee. In all cases, consent for research was obtained from the donor family and organ procurement organization. Eight paired human kidneys from four donors deemed unsuitable for transplantation after being offered and declined were included in this study and transported to our research lab at the University of California Davis Health System. After an initial period of cold storage, two paired kidneys (kidney ID 3 and 4) were placed on HMP (RM3 System; Waters Medical Systems, Rochester, MN) using Kidney Perfusion Solution-1 (Organ Recovery System, Itasca, IL) with 40 international units of insulin and 19 ml of 20% mannitol with the intention to proceed with transplantation (Figure 1A).

### EVNP System

All kidneys were placed on an EVNP system using a pediatric cardiopulmonary bypass system, as previously described.^2^ Once connected to the circuit, flow bioprobe (model TX50P; Medtronic) and pressure monitors (model 66000; Medtronic) were used to continuously perfuse the kidneys with a mean arterial pressure of 70–80 mm Hg for 3 hours at a temperature of 37°C. Flow was adjusted to keep pressure between the set points.

One kidney from each pair was randomly assigned to either WB or PRBC EVNP and perfused with a compatible, group-matched PRBC-based or WB-based perfusate (Figure 1B). The PRBC perfusate consisted of leukocyte and platelet depleted, washed red cell unit blood O positive, which were acquired from our internal hospital blood bank and diluted in a 1:1 ratio with Plasmalyte-A (Baxter Medical, Deerfield, IL). The WB perfusate was type-specific blood in citrate phosphate dextrose adenine solution, collected 24 hours prior from the local blood center laboratory from repeated male donors with no history of infectious diseases stored at room temperature (Bloodsource, Mather, CA). The circuit was primed by recirculation of the prepared PRBC or WB perfusate oxygenated with 95% O_2_/5% CO_2_ and supplemented with heparin sodium (2000 international units; NOVAPLUS) and exogenous anhydrous creatinine (0.06 g; MP Biomedicals, Burlingame, CA) to maintain physiologic levels. In addition, the EVNP circuit was primed with 500 ml of this perfusate supplemented with 20 ml of parenteral nutrition (Baxter CLINIMIX E 2.75/10), which was infused with 100 units of regular insulin (Humulin R), 5 ml of multivitamins (Baxter Medical), and 26 ml of 8.4% bicarbonate (Millipore Sigma, St. Louis, MO) per liter of solution to keep perfusate within a 7.3–7.4 pH range. This solution was continued at the rate of 20 ml/h until the end of the perfusion period. Urine loss was replaced at a 1:1 ratio with Plasmalyte-A for perfusate solutions.

### Kidney Assessment

Hemodynamic parameters (pressure and flow) were recorded every 30 minutes, and renal resistance (RR) was calculated as pressure (mm Hg)/flow (ml/min). Urine was collected every 30 minutes and stored in for further analysis. Urine samples were later analyzed for sodium using a Critical Care Xpress Machine (Nova Biomedical, Waltham, MA) and for creatinine using a creatinine parameter assay kit (R&D systems, Minneapolis, MN) for calculation of creatinine clearance and fractional excretion of sodium (FENa), as reported previously.^2^

Perfusate samples were collected every 30 minutes from a port in the arterial line and directly from the venous return. The samples were analyzed by a Critical Care Xpress machine for pH, pCO_2_, and electrolytes, and handheld point of care devices (Stat sensors; Nova Biomedical) for creatinine, lactate, and glucose levels. A wedge biopsy of the renal cortex was collected at the end of the perfusion for histology.

### Urinary AKI Biomarkers

Urinary neutrophil gelatinase-associated lipocalin (NGAL) was measured with a point of care fluorescence immunoassay system (Bio Site Triage MeterPro Alere, Waltham, MA). Urinary kidney injury molecule-1 (KIM-1) was measured with a human urinary KIM-1 Quantikine ELISA kit per manufacturer's instructions (R&D Systems).

### Metabolomics and Lipid Profile

Renal cortical core tissue needle biopsies (16 g Tru Core II Biopsy Instrument; Argon Medical Devices, Athens, TX) were taken before and after EVNP and snap frozen in liquid nitrogen. After study completion, samples (*n*=16) were submitted to the West Coast Metabolomics Center at the University of California Davis Genome Center. Samples were analyzed with validated chromatography-mass spectrometry methods with 15–25 internal standards, for a nontargeted primary metabolites analysis. Lipidomics profiling was performed using liquid chromatography coupled to quadrupole time-of-flight mass spectrometer-charged surface hybrid (LC QTOF CHS). A total of 185 metabolites and 220 lipid species were identified across all eight kidneys.

### Statistical Analysis

We used both *t* test and the area under the curve measurements (using the trapezoid rule) to assess and validate group differences in renal blood flow (RBF), RR, urine output (UO), perfusate creatinine, perfusate lactic acid, urinary NGAL, urinary KIM-1, and calculated FENa during EVNP. In addition, we fit a linear mixed-effects model to compare changes over time during the 3-hour EVNP. These models included a random effect for intercept and slope to accommodate for differences at baseline levels, dependency within matched pairs, and the repeated measures over time. We used ANOVA adjusted for multiple hypothesis testing (Bonferroni) to determine significant differences in hemodynamics and metabolites over time. The means and SEM are provided. We used a paired *t* test to assess within group tissue temporal metabolic and lipid profile changes. Similarly, baseline metabolic and lipid profile differences were assessed using a paired *t* test. To assess temporal tissue metabolic and lipid profile differences between the two groups, a two-way ANOVA using the interaction of time (pre versus post) and group (PRBC versus WB) was used. MetaboAnalyst 6.0 was used for enrichment analysis. All tests were two-sided and *P* < 0.05 was concluded statistically significant. Analyses were conducted in R version 4.2.2.

## Results

### Donor Demographics and Characteristics of Kidneys

The mean donor age was 61 years (range 54–67 years), and the mean donor weight was 92 kg (range 66–137 kg) (Table 1). There was one donation after cardiac death donor and three extended criteria donor donors with a mean kidney donor profile index of 87% (range 77%–99%). Kidneys were discarded due to a combination of donor history, cold ischemia time (CIT), and biopsy results. Four of the paired kidneys were placed on HMP before discard for a mean time of 4 hours (Figure 1A and Table 1).

**Table 1:** Donor demographics and characteristics of kidneys placed on *ex vivo* normothermic perfusion

Kidney ID	1		2		3		4	
Age (yr)	67		58		54		64	
M/F	F		M		M		F	
HTN	Yes		No		No		Yes	
DM	Yes		No		No		Yes	
Weight (kg)	74		137		66		91	
Cause of death	Anoxia		CVA		Anoxia		CVA	
Terminal creatinine (mg/dl)	1.70		3.40		1.66		0.55	
KDPI (%)	99		79		75		94	
ECD or SCD	ECD		ECD		SCD		ECD	
DBD or DCD	DBD		DBD		DCD		DBD	
Total CIT (h)	40	41	54	55	56	56	63	64
Static cold storage (h)	40	41	54	55	50	49	62	63
HMP (h)	0	0	0	0	6	7	1	1
Anatomical position	Left	Right	Right	Left	Left	Right	Left	Right
Biopsy results	17% GS	19% GS	19% GS	20% GS	12% GS	14% GS	0% GS	0% GS
	Mild IF	Mild IF	Mild to Mod IF	Mild to Mod IF	Mild IF	Mild IF	Mild IF	Mild IF
	Mild TA	Mild TA	Mild TA	Mild to Mod TA	Mild TA	Mild TA	Mild TA	Mild TA
	Mild AS	Mild AS	Mod to severe AS	Mod to severe AS	Mod AS	Mod AS	Severe AS	Mod to severe AS
					Benign cortical cyst			
EVNP perfusate	WB	PRBC	WB	PRBC	WB	PRBC	WB	PRBC

AS, arteriolosclerosis; CIT, cold ischemia time; CVA, cerebrovascular accident; DBD, donation after brain death; DCD, donation after cardiac death; DM, diabetes mellitus; ECD, extended criteria donor; EVNP, *ex vivo* normothermic perfusion; F, female; GS, glomerulosclerosis; HMP, hypothermic machine perfusion; HTN, hypertension; IF, interstitial fibrosis; KDPI, kidney donor profile index; M, male; PRBC, packed red blood cell; SCD, standard criteria donor; TA, tubular atrophy; WB, whole blood.

### Hemodynamics and Functional Assessment

**Figure 2:**
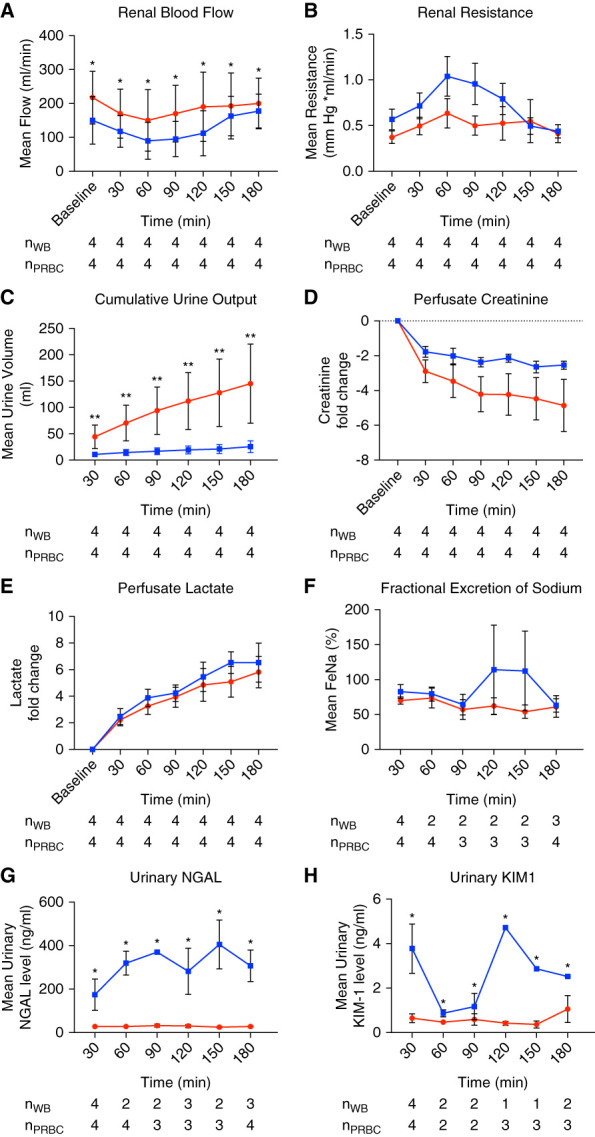
**Hemodynamic and functional parameters as well as urinary biomarkers are compared over time within and between perfusate groups.** (A) renal blood flow, (B) intrarenal resistance, (C) cumulative urine output, (D) perfusate creatinine, (E) lactate, (F) fraction excretion of sodium, (G) concentration of urinary NGAL, and (H) KIM1. Graphs are plotted as mean, and error bars represent SEM. The blue lines represent the WB group, and the red lines represent the PRBC group. The number of available data for each group at a given time point is shown at the bottom of each panel. *P < 0.05; **P < 0.001. FENa, fractional excretion of sodium; KIM-1, kidney injury molecule-1; NGAL, neutrophil gelatinase-associated lipocalin; PRBC, packed red blood cell; WB, whole blood.

All kidneys exhibited a healthy pink color after the initiation of EVNP. The PRBC perfused kidney group initially had a higher mean RBF of 217 (77) ml/min compared with an initial flow of 150 (70) ml/min in the WB perfused kidney group and maintained a higher RBF throughout the perfusion period (Figure 2A and Table 2). RBF decreased in both groups to a nadir at 1 hour, after which flow increased gradually thereafter until the end of perfusion period. Overall, RR was lower among the PRBC group, initially increasing over the first hour, followed by a gradual decrease for both groups (Figure 1B and Table 2). Total UO volume after EVNP was significantly higher in the PRBC group compared with the WB group (Figure 2C and Table 2).

**Table 2:** Summary of hemodynamics, functional, and injury biomarkers comparing whole blood and packed red blood cell at baseline and postperfusion

Outcomes	Baseline		Postperfusion		Between Group Differences	*P* Value
	WB	PRBC	WB	PRBC	PRBC-WB (95% CI)	
RBF, ml/min	150 (70)	217 (77)	177 (50)	200 (75)	55 (21 to 89)	0.004
RR, mm Hg × ml/min	0.57 (0.23)	0.37 (0.13)	0.44 (0.14)	0.41 (0.19)	−0.21 (−0.42 to −0.01)	0.037
UO, ml	10.7 (8.3)	44.4 (45)	25.5 (22)	145.1 (151)	81 (47 to 115)	<0.001
Perfusate creatinine, fold change	−1.8 (0.6)	−2.9 (1.3)	−2.5 (0.4)	−4.8 (3)	1.5 (−0.03 to 3.1)	0.056
Perfusate lactate, fold change	2.5 (1.2)	2.2 (0.8)	6.5 (2.9)	5.8 (2.3)	−0.6 (−2.6 to 1.2)	0.45
Fraction excretion of sodium, %	83 (20)	70 (10)	63 (16)	61 (31)	−23 (−44.4 to −1.4)	0.038
Creatinine clearance, ml/min	0.15 (0.13)	0.87 (0.83)	0.25 (0.19)	2.3 (3.8)	1.3 (0.8 to 1.7)	<0.001
Urinary NGAL, ng/ml	173 (144)	27 (12)	306 (127)	27 (4)	−281 (−354 to −208)	<0.001
Urinary KIM-1, ng/ml	3.8 (2.2)	0.64 (0.38)	2.5 (0.03)	1.05 (1.06)	−2.1 (−3.4 to −0.7)	0.007

Baseline values for urine output, perfusate creatinine and lactate, fractional excretion of sodium, creatinine clearance, urinary neutrophil gelatinase-associated lipocalin, and kidney injury molecule-1 represent 30-minute after the initiation of perfusion. The values represent mean and SD. CI, confidence interval; KIM-1, kidney injury molecule-1; NGAL, neutrophil gelatinase-associated lipocalin; PRBC, packed red blood cell; RBF, renal blood flow; RR, renal resistance; UO, urine output; WB, whole blood.

Overall, perfusate creatinine decreased by a mean fold change of 3.7 over three in both groups but declined faster in the PRBC group compared with the WB group (Figure 2D and Table 2). Perfusate lactic acid levels increased significantly in the WB and PRBC groups at a similar rate (Figure 2E and Table 2). The PRBC perfused kidneys had a more stable and 23% lower FENa when compared with the WB perfused kidneys (Figure 2F and Table 2). Creatinine clearance was also higher in the PRBC kidneys over time than the WB kidneys (Table 2).

### Kidney Biomarkers Trajectories

Urinary NGAL concentrations were significantly lower in the PRBC perfused kidneys compared with the WB perfused kidneys (Figure 2G and Table 2). The WB perfused kidneys also exhibited a progressive increase trend in urinary NGAL levels over the perfusion period when compared with the PRBC perfused group. Urinary KIM-1 concentrations also showed statistically lower values and a more stable trend in the PRBC perfused kidneys than the WB perfused kidneys which had fluctuating values throughout the perfusion period (Figure 2H and Table 2).

### Histology

**Figure 3:**
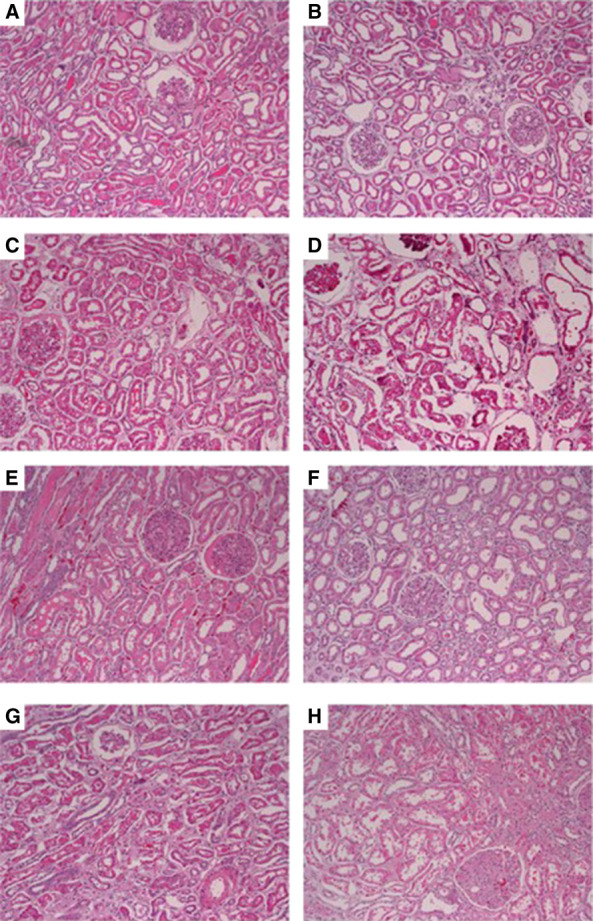
**Histology after 3 hours of EVNP (100×).** Renal cortical biopsies: hematoxylin and eosin stained, after 3 hours of *ex vivo* normothermic perfusion with either whole blood (A, C, E, and G) or the paired kidney perfused with packed red blood cell–based solution (B, D, F, and H, respectively). All kidneys showed widespread acute tubular injury with flattening of the tubular epithelia, dilation of tubular lumina, and irregular vacuolization of the tubular epithelial cytoplasm. Scattered tubules contained tubular epithelial cells and cell debris. Associated mild interstitial edema is noted as well. Images were captured at 100× magnification.

All biopsies taken at the end of the perfusion period showed widespread acute tubular injury with flattening of the tubular epithelia, dilation of tubular lumina, and irregular vacuolization of the tubular epithelial cytoplasm with no meaningful differences noted between the WB and PRBC perfused kidneys (Table 1). Associated mild interstitial edema and scattered tubular cellular debris was noted as well (Figure 3).

### Tissue Metabolic Changes

**Figure 4:**
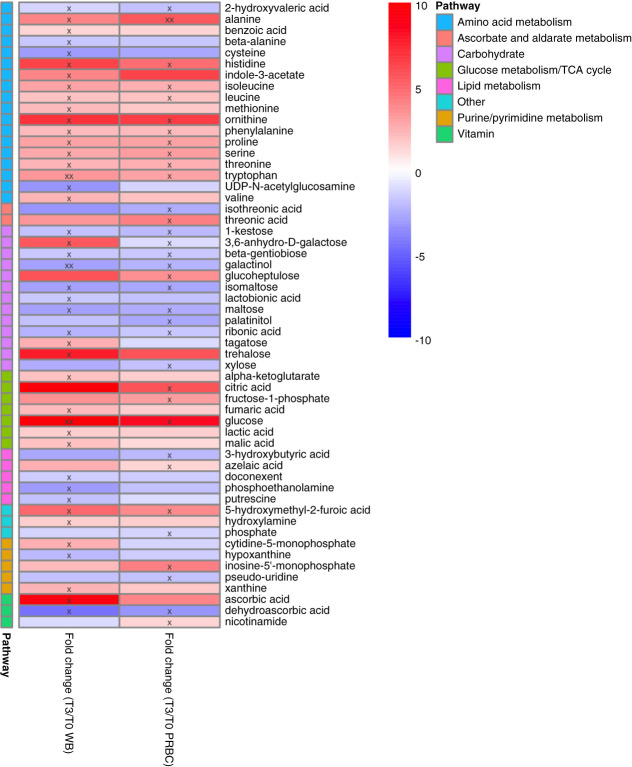
**Tissue metabolic changes comparing preperfusion versus postperfusion in the WB (*n*=4) and PRBC (*n*=4) groups.** The fold changes are in reference to baseline. “x” represents a *P* < 0.05 and “xx” represents *P* < 0.001.

Before the initiation of perfusion tissue, metabolic profile differences were minimal (1%) between groups. We observed marked tissue metabolic alterations in the WB group over time with a total of 43 (23%) metabolites compared with baseline (Figure 4). These temporal differences were characterized by heightened levels of (branched chain) amino acids (13/43 altered metabolites) and metabolites involved in mitochondrial energy metabolism. The metabolites with the largest fold changes were glucose (9.8), ascorbic acid (9.2), and trehalose (7.6) (Figure 3 and Supplemental Table 1). Pathway analysis revealed amino acid and carbohydrate metabolism and TCA cycle as the main altered metabolic pathways in the WB group during the 3-hour perfusion (Supplemental Figure 1A).

In the PRBC group, temporal tissue metabolic differences were noted for a total of 34 (18%) significant alteration comparing preperfusion and postperfusion (Figure 4). Similar to the WB group, temporal differences were associated with elevated tissue levels of amino acids (10/34 altered metabolites) and carbohydrates. The metabolites with the largest fold changes over time were glucose (8.3), ornithine (6.4), and citric acid (5.6) (Figure 4 and Supplemental Table 2). Pathway analysis highlighted similar altered metabolic pathways to the WB group identifying (branched chain) amino acids and carbohydrate metabolism as the predominant altered pathways over time (Supplemental Figure 1B).

**Figure 5:**
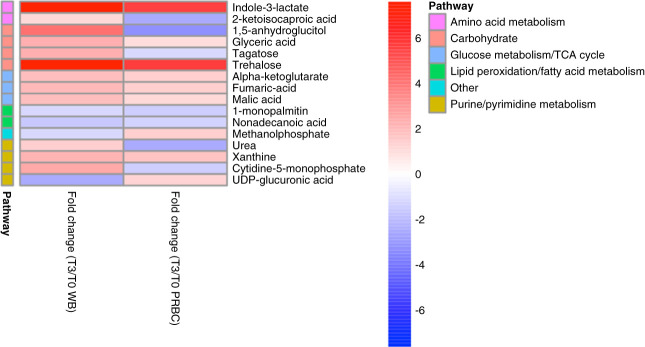
**Tissue metabolic differences comparing WB (*n*=4) and PRBC (*n*=4) over time.** The fold changes are in reference to baseline. Metabolites with interaction *P* < 0.05 are shown.

We found a remarkable overlap in the significantly altered tissue metabolites over time regardless of perfusion condition (WB or PRBC). Overall, changes in 20 metabolites were overlapping in both groups; however, the magnitude of differences was less pronounced in the PRBC compared with the WB group (Figure 4). The overlap highlighted aberrations involving amino acids and carbohydrate metabolism. In addition, we identified differences in tissue metabolic changes from baseline to postperfusion comparing both groups. We found a total of 16 tissue metabolites to be significantly different comparing WB and PRBC groups over time (Figure 5). These metabolites included purine/pyrimidine metabolites, TCA cycle intermediates, and lipid peroxidation products. Metabolic pathways distinguishing the metabolic changes between the groups were amino acid (arginine, alanine, aspartate, and glutamate) metabolism, TCA cycle, and pyruvate metabolism (Supplemental Figure 1C).

### Tissue Lipid Profile Changes

In contrast to the minimal baseline difference in tissue metabolic profile between WB and PRBC groups, we noted meaningful baseline lipid profile differences including 34 (16%) significantly different lipid species distinguishing WB and PRBC groups (Supplemental Figure 2). All significantly different lipid species were lower in the WB group compared with PRBC. These lipid species were predominantly composed of glycerophospholipids, followed by glycerolipids (TGs) and sphingolipids (Supplemental Table 1).

**Figure 6:**
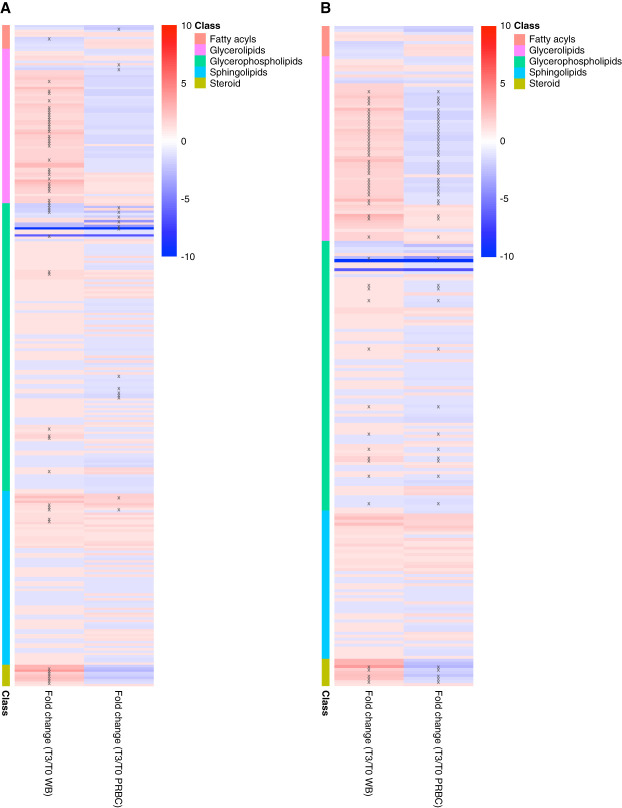
**Tissue lipid profile differences pre and post perfusion within WB (*n*=4) and PRBC (*n*=4) groups and over time group comparisons.** (A) Tissue lipid profile within each group comparing preperfusion and postperfusion. The fold changes are in reference to baseline. “x” represents a *P* < 0.05 and “xx” represents *P* < 0.001. (B) Tissue lipid profile differences comparing WB and PRBC over time. Lipids with interaction *P* < 0.05 are shown.

Within-group temporal lipid profile assessment revealed marked lipid species alterations in the WB group. Compared with baseline, a total of 48 (22%) tissue lipid species were significantly different postperfusion (Figure 6). Most of the altered tissue lipids were elevated postperfusion compared with baseline levels. These lipids were mainly plasma membrane/structure components such as TGs, glycerophospholipids, and steroids (Figure 5 and Supplemental Table 3).

The PRBC group demonstrated substantially fewer temporal differences in tissue lipid profile compared with the WB group. Only 13 (6%) of lipid species were significantly altered comparing preperfusion and postperfusion (Figure 6). They predominantly consisted of glycerophospholipids. Interestingly, most of the altered lipids were lower postperfusion compared with baseline (Figure 5 and Supplemental Table 4).

A total of 53 (24%) lipid species were significantly different between the WB and PRBC groups over time. Unlike the PRBC group, most of these lipid species were elevated postperfusion among the kidneys in the WB group (Figure 6 and Supplemental Table 5).

## Discussion

Despite the use of currently available modalities, many high-risk kidneys are discarded due to the inability to adequately assess the risk of poor outcomes before transplantation. Here, we used a 3-hour EVNP system to assess hemodynamics, functional, and tissue metabolic changes among eight paired high-risk deceased donor kidneys with relatively long CIT using PRBC or WB. Despite the similarity of WB and PRBC perfused kidneys in histology, kidneys perfused with PRBC had higher urine production and RBF and lower levels of urinary NGAL and KIM-1, suggesting improved viability. Metabolomics assessment during EVNP showed more drastic metabolic aberration in the WB group involving amino acid, carbohydrate, and mitochondrial energy metabolism. Tissue lipid profiling showed that WB was associated with accumulation of tissue membrane/structure components including TGs, ceramides, and cholesteryl esters postperfusion compared with the PRBC which demonstrated a minimal lipid profile change over time. Together, our findings reveal the superiority of PRBCs (leukocyte and platelet depletion) in facilitating and maintaining functional and metabolic recovery of marginal kidneys during an EVNP.

Our findings are consistent with previous studies showing that compared with WB leukocyte and platelet depleted blood based perfusate improved postischemic renal function after a period of warm ischemia.^20,21^ Consistent with the studies from Hosgood,^20,21^ we observed that the kidneys perfused with a PRBC perfusate exhibit superior kidney functional parameters compared the WB perfused kidneys evident by higher RBF indicating superior vascular integrity (lower vascular injury and interstitial edema). We also observed evidence of higher glomerular filtration and tubular function with higher UO, decreasing perfusate creatinine, and a lower fraction excretion of sodium of the PRBC group. Finally, urinary inflammatory mediators and markers of proximal tubule injury were stable and consistently lower among the PRBC group compared with WB. Overall, our findings corroborate previous studies showing the role of leukocytes and platelets in exacerbating early kidney reperfusion injury resulting in impaired kidney function.

The relatively greater kidney function in the PRBC perfusate group compared with WB was observed in the absence of significant histologic differences between the two groups. In both groups, we observed comparable histologic damage associated with reperfusion injury resulting in increased tubular dilation and vacuolation. Two studies comparing porcine kidney perfusion using WB and PRBC have shown similar findings. Harper *et al.* showed no meaningful histologic difference even after 6 hours of normothermic perfusion comparing WB and PRBC.^20,21^ This suggests that leukocyte and platelet depletion may not be sufficient in mitigating tubular damage induced by early graft reperfusion as reported by others.^20,21^ It is also possible that tubular damage occurs through a different mechanism than vascular damage. Overall, our short-term postperfusion histologic assessment did not agree with functional differences and may not be a sensitive early time point indicator for kidney function and recovery potential for high-risk kidneys.

Tissue metabolic profiling of the renal cortex has the advantage of providing biologic insight into the metabolic basis for kidney reperfusion injury, the influence of presence of leukocytes and platelets on metabolism during perfusion, and the association of metabolic health with kidney function. First, we found notable similarities in within-group temporal changes among both groups involving impairment in amino acids (and branched chain amino acids [BCAAs]) and glucose metabolism; however, these changes were more pronounced (higher fold changes of the same metabolites) among the WB group. Regardless of perfusion conditions, ischemia reperfusion injury is accompanied by metabolic aberrations associated and the presence of leukocytes may further influence glucose and amino acid metabolism. The proximal tubule actively reabsorbs nearly all the filtered glucose and amino acids, incurring a high energetic demand from mitochondrial oxidative phosphorylation.^25,26^ Second, the WB group was associated with diminished mitochondrial energy metabolism evidenced by impaired TCA cycle and pyruvate metabolism. Third, the differences in tissue metabolic profile were reflected in the hemodynamic and functional differences among the two groups. These findings are consistent with our previous study reporting that BCAA and glucose utilization are major determinants of kidney function and recovery in high-risk kidneys perfused using the same EVNP system.^27^ Our findings also agree with another study that identified “amino acid transport” as one of the top altered metabolic pathways associated with DGF from a cohort of 190 kidneys transplanted posthypothermic perfusion.^28^ Together, our data suggest that leukocyte depleted perfusion of high-risk kidneys might mitigate reperfusion-associated metabolic derangements linked to mitochondrial dysfunction.

Leveraging practical and easy-to-measure tissue metabolic markers and simultaneous functional measures in the *ex vivo* setting allows for a more comprehensive pretransplant organ assessment approach to predict long-term graft function and determine suitability for transplantation. In addition to amino acids and TCA cycle intermediates, we identified other tissue metabolites of interest that warrant further examination and validation to be used as such biomarkers. These metabolites include 1, 5 anhydroglucitol (1, 5 AG), tagatose, and 2-ketoisocaproic acid, which were all elevated compared with their baseline level in the WB group. The clinical relevance of 1, 5 AG was recently underscored by a longitudinal analysis of 1612 adults with mean eGFR of 62 ml/min per 1.73 m^2^ from the Atherosclerosis Risk in Communities study showing that serum 1, 5 AG were associated with 40% decline in eGFR.^29^ In addition, tagatose has also been identified as a sensitive and specific metabolite for early diagnosis of acute ejection fraction after heart transplantation in preclinical models.^30^ Finally, 2-ketoisocaproic acid, the (abnormal) catabolic product of leucine catalyzed by the enzyme branched-chain alpha-keto acid dehydrogenase complex,^31^ may serve as a biomarker for impaired BCAA metabolism and mitochondrial health as is it localized in the inner mitochondrial membrane.

Tissue lipid profiling revealed marked differences between the WB and PRBC groups. We found more extensive lipid profile differences among the WB group. These lipids involved significant accumulation of plasma membrane/structure components such as TGs, sphingolipids (ceramides), and steroids (cholesteryl esters). This elevation in cell membrane components suggests that increased tissue damage and necrosis resulted from ischemia damage in the presence of leukocytes and platelets. This is in line with our recent study showing early accumulation of plasma membrane lipids, including TGs and sphingolipids, linked with poor kidney function and recovery during a 12-hour EVNP perfusion period.^27^ This is also supported by another study showing that fatty acid accumulation during HMP is significantly associated with development of death-censored graft failure.^28^ Mechanistic studies support leukocyte depletion shown to reduce tubular apoptosis, caspase-3 activity, and IL-1*β* activation in porcine kidneys.^22^ Together, our findings suggest that the presence of leukocytes might further exacerbate ischemia injury contributing to poor kidney function limiting recovery potential.

Our study had notable strengths and limitations. Using a well-established EVNP system, we performed a comprehensive organ assessment consisting of hemodynamic and functional measurements, histologic analysis, and untargeted tissue metabolic/lipid profiling of high-risk kidneys. We evaluated eight paired kidneys from four individuals allowing us to minimize between group variations in our assessments. Indeed, our study was not without limitations. Despite using eight paired kidneys, our study cohort size was relatively small. The long CIT of the kidneys (>50 hours) is at the extreme limit that many transplant centers would consider acceptable for transplantation. The perfusion duration was only limited to 3 hours, and it is possible that a longer perfusion period is necessary to adequately assess kidney viability and validate longer-term post-transplant outcomes. Finally, lower viability combined with reduced clearance among the WB group might have led to passive diffusion of metabolites into the cells contributing to exaggerated tissue metabolite levels over time.

In conclusion, this study supports the potential role of leukocyte depleted EVNP system facilitating recovery of high-risk kidneys that are currently being discarded. There are currently no widely accepted EVNP criteria that definitively determine which high-risk kidneys are suitable for transplantation. In addition to the previously established criteria for kidney transplantation such as macroscopic appearance, RBF, and urine production^32,33^; early time point tissue metabolic and lipid profile biomarkers (TGs) should also be considered to enhance the predictability and success of the pretransplant criteria. In addition, longer duration studies with more comprehensive metabolic substrates are needed to fully assess the potential of the EVNP system in recovering marginal kidneys.

## Supplementary Material

## Data Availability

Anonymized data created for the study are or will be available in a persistent repository upon publication. Analyzable Data. Figshare. A complete deidentified metadata supporting the findings in this study has been made available on Figshare (DOI: 10.6084/m9.figshare.26961916). Tissue metabolomics and lipidomics data can be found at (DOI: 10.6084/m9.figshare.26961934). Additional information will be made available to share upon request.

## Supplemental Material

Supplemental Table 1. Tissue temporal metabolic differences among the WB group comparing preperfusion and postperfusion (*n*=4).

Supplemental Table 2. Tissue temporal metabolic differences among the PRBC group comparing preperfusion and postperfusion (*n*=4).

Supplemental Table 3. Tissue temporal lipid profile differences among the WB group comparing preperfusion and postperfusion (*n*=4).

Supplemental Table 4. Minimal temporal differences in tissue lipid profile among PRBC perfused kidneys (*n*=4).

Supplemental Table 5. Differences in tissue lipid profile comparing WB (*n*=4) and PRBC (*n*=4) over time.

Supplemental Figure 1. Metabolic pathway analysis of changes within WB/PRBC groups and temporal derangements comparing the two groups. (A) Pre-WB versus post-WB, (B) pre-PRBC versus post-PRBC, and (C) WB versus PRBC over time. The size and color of the nodes represent pathway impact value and *P* value, respectively.

Supplemental Figure 2. Tissue lipid baseline differences comparing WB and PRBC. “x” represents a *P* < 0.05 and “xx” represents *P* < 0.001.
