# Supplementary material for: Packed Red Blood Cell and Whole Blood Perfusates during Ex Vivo Normothermic Perfusion for Assessment of High-Risk Donor Kidneys
Source: Kidney360. 2025 May 7;6(9):1573–85. doi: 10.34067/KID.0000000815 (PMC12503136; doi:10.34067/KID.0000000815)
Supplement: Supplementary file 1 [file kidney360-6-01573-s001.pdf]

ASN
American Society of Nephrology
JASN
Journal of the American Society of Nephrology
KIDNEY360
Accessing Our World From Every Angle
CJASN
Clinical Journal of the American Society of Nephrology

ASN Journal Disclosure Form

As per ASN journal policy, I have disclosed any financial relationships or commitments I have held in the past 36 months as included below. I have listed my Current Employer below to indicate there is a relationship requiring disclosure. If no relationship exists, my Current Employer is not listed.

A. Ahmadi reports the following:
Employer: University of California San Diego

I understand that the information above will be published within the journal article, if accepted, and that failure to comply and/or to accurately and completely report the potential financial conflicts of interest could lead to the following: 1) Prior to publication, article rejection, or 2) Post-publication, sanctions ranging from, but not limited to, issuing a correction, reporting the inaccurate information to the authors' institution, banning authors from submitting work to ASN journals for varying lengths of time, and/or retraction of the published work.

Name: Armin Ahmadi
Manuscript ID: K360-2025-000015R1
Manuscript Title: Packed Red Blood Cell and Whole Blood Perfusates during Ex-vivo Normothermic Perfusion for Assessment of High-Risk Donor Kidneys
Date of Completion: March 10, 2025
Disclosure Updated Date: March 10, 2025

ASN American Society of Nephrology | JASN Journal of the American Society of Nephrology | KIDNEY360 Accessing Our World From Every Angle | CJASN Clinical Journal of the American Society of Nephrology

ASN Journal Disclosure Form

As per ASN journal policy, I have disclosed any financial relationships or commitments I have held in the past 36 months as included below. I have listed my Current Employer below to indicate there is a relationship requiring disclosure. If no relationship exists, my Current Employer is not listed.

S. Fan reports the following:
Employer: ctsc

I understand that the information above will be published within the journal article, if accepted, and that failure to comply and/or to accurately and completely report the potential financial conflicts of interest could lead to the following: 1) Prior to publication, article rejection, or 2) Post-publication, sanctions ranging from, but not limited to, issuing a correction, reporting the inaccurate information to the authors' institution, banning authors from submitting work to ASN journals for varying lengths of time, and/or retraction of the published work.

Name: Sili Fan
Manuscript ID: K360-2025-000015R1
Manuscript Title: Packed Red Blood Cell and Whole Blood Perfusates during Ex-vivo Normothermic Perfusion for Assessment of High-Risk Donor Kidneys
Date of Completion: March 10, 2025
Disclosure Updated Date: March 10, 2025

ASN American Society of Nephrology · JASN Journal of the American Society of Nephrology · KIDNEY360 Accessing Our World From Every Angle · CJASN Clinical Journal of the American Society of Nephrology

ASN Journal Disclosure Form

As per ASN journal policy, I have disclosed any financial relationships or commitments I have held in the past 36 months as included below. I have listed my Current Employer below to indicate there is a relationship requiring disclosure. If no relationship exists, my Current Employer is not listed.

N. Goussous reports the following:
Employer: UC Davis

I understand that the information above will be published within the journal article, if accepted, and that failure to comply and/or to accurately and completely report the potential financial conflicts of interest could lead to the following: 1) Prior to publication, article rejection, or 2) Post-publication, sanctions ranging from, but not limited to, issuing a correction, reporting the inaccurate information to the authors' institution, banning authors from submitting work to ASN journals for varying lengths of time, and/or retraction of the published work.

Name: Naeem Goussous
Manuscript ID: K360-2025-000015R1
Manuscript Title: Packed Red Blood Cell and Whole Blood Perfusates during Ex-vivo Normothermic Perfusion for Assessment of High-Risk Donor Kidney
Date of Completion: March 7, 2025
Disclosure Updated Date: March 7, 2025

ASN American Society of Nephrology | JASN Journal of the American Society of Nephrology | KIDNEY360 Accessing Our World From Every Angle | CJASN Clinical Journal of the American Society of Nephrology

ASN Journal Disclosure Form

As per ASN journal policy, I have disclosed any financial relationships or commitments I have held in the past 36 months as included below. I have listed my Current Employer below to indicate there is a relationship requiring disclosure. If no relationship exists, my Current Employer is not listed.

K. Jen reports the following:
Advisory or Leadership Role: Novartis; and Speakers Bureau: Alexion.

I understand that the information above will be published within the journal article, if accepted, and that failure to comply and/or to accurately and completely report the potential financial conflicts of interest could lead to the following: 1) Prior to publication, article rejection, or 2) Post-publication, sanctions ranging from, but not limited to, issuing a correction, reporting the inaccurate information to the authors' institution, banning authors from submitting work to ASN journals for varying lengths of time, and/or retraction of the published work.

Name: Kuang-Yu Jen
Manuscript ID: K360-2025-000015R1
Manuscript Title: Packed Red Blood Cell and Whole Blood Perfusates during Ex-vivo Normothermic Perfusion for Assessment of High-Risk Donor Kidneys
Date of Completion: March 10, 2025
Disclosure Updated Date: May 17, 2024

ASN
American Society of Nephrology
JASN
Journal of the American Society of Nephrology
KIDNEY360
Accessing Our World From Every Angle
CJASN
Clinical Journal of the American Society of Nephrology

ASN Journal Disclosure Form

As per ASN journal policy, I have disclosed any financial relationships or commitments I have held in the past 36 months as included below. I have listed my Current Employer below to indicate there is a relationship requiring disclosure. If no relationship exists, my Current Employer is not listed.

I. Palma reports the following:
Employer: University of California-Davis

I understand that the information above will be published within the journal article, if accepted, and that failure to comply and/or to accurately and completely report the potential financial conflicts of interest could lead to the following: 1) Prior to publication, article rejection, or 2) Post-publication, sanctions ranging from, but not limited to, issuing a correction, reporting the inaccurate information to the authors' institution, banning authors from submitting work to ASN journals for varying lengths of time, and/or retraction of the published work.

Name: Ivonne Palma
Manuscript ID: K360-2025-000015R1
Manuscript Title: Packed Red Blood Cell and Whole Blood Perfusates during Ex-vivo Normothermic Perfusion for Assessment of High-Risk Donor Kidneys
Date of Completion: March 10, 2025
Disclosure Updated Date: March 10, 2025

ASN
American Society of Nephrology
JASN
Journal of the American Society of Nephrology
KIDNEY360
Accessing Our World From Every Angle
CJASN
Clinical Journal of the American Society of Nephrology

ASN Journal Disclosure Form

As per ASN journal policy, I have disclosed any financial relationships or commitments I have held in the past 36 months as included below. I have listed my Current Employer below to indicate there is a relationship requiring disclosure. If no relationship exists, my Current Employer is not listed.

R. Perez reports the following:
Employer: University of California, Davis

I understand that the information above will be published within the journal article, if accepted, and that failure to comply and/or to accurately and completely report the potential financial conflicts of interest could lead to the following: 1) Prior to publication, article rejection, or 2) Post-publication, sanctions ranging from, but not limited to, issuing a correction, reporting the inaccurate information to the authors' institution, banning authors from submitting work to ASN journals for varying lengths of time, and/or retraction of the published work.

Name: Richard V. Perez
Manuscript ID: K360-2025-000015R1
Manuscript Title: Packed Red Blood Cell and Whole Blood Perfusates during Ex-vivo Normothermic Perfusion for Assessment of High-Risk Donor Kidneys
Date of Completion: April 10, 2025
Disclosure Updated Date: April 10, 2025

ASN American Society of Nephrology
JASN Journal of the American Society of Nephrology
KIDNEY360 Accessing Our World From Every Angle
CJASN Clinical Journal of the American Society of Nephrology

ASN Journal Disclosure Form

As per ASN journal policy, I have disclosed any financial relationships or commitments I have held in the past 36 months as included below. I have listed my Current Employer below to indicate there is a relationship requiring disclosure. If no relationship exists, my Current Employer is not listed.

B. Roshanravan reports the following:
Employer: University of California Davis; VA Healthcare System; and Other Interests or Relationships: Deputy Editor Role for the Clinical Journal of the American Society of Nephrology (CJASN).

I understand that the information above will be published within the journal article, if accepted, and that failure to comply and/or to accurately and completely report the potential financial conflicts of interest could lead to the following: 1) Prior to publication, article rejection, or 2) Post-publication, sanctions ranging from, but not limited to, issuing a correction, reporting the inaccurate information to the authors' institution, banning authors from submitting work to ASN journals for varying lengths of time, and/or retraction of the published work.

Name: Baback Roshanravan
Manuscript ID: Manuscript ID (K360-2025-000015R1)
Manuscript Title: Packed Red Blood Cell and Whole Blood Perfusates during Ex-vivo Normothermic Perfusion for Assessment of High-Risk Donor Kidneys
Date of Completion: April 9, 2025
Disclosure Updated Date: April 9, 2025

ASN American Society of Nephrology
JASN Journal of the American Society of Nephrology
KIDNEY360 Accessing Our World From Every Angle
CJASN Clinical Journal of the American Society of Nephrology

ASN Journal Disclosure Form

As per ASN journal policy, I have disclosed any financial relationships or commitments I have held in the past 36 months as included below. I have listed my Current Employer below to indicate there is a relationship requiring disclosure. If no relationship exists, my Current Employer is not listed.

J. Sageshima reports the following:
Employer: University of California, Davis; Ownership Interest: AAPL; ABNB; AI; AMZN; COST; DDOG; DIS; ETSY; EVER; GOOG; INTC; LUV; LYFT; MGA; MSFT; NVDA; OKTA; PLTR; PLUG; PYPL; QCOM; SHOP; SNOW; SOFI; SON; SPCE; SQ; TSLA; TM; U; VIGAX; ZM; and Research Funding: ITB-MED.

I understand that the information above will be published within the journal article, if accepted, and that failure to comply and/or to accurately and completely report the potential financial conflicts of interest could lead to the following: 1) Prior to publication, article rejection, or 2) Post-publication, sanctions ranging from, but not limited to, issuing a correction, reporting the inaccurate information to the authors' institution, banning authors from submitting work to ASN journals for varying lengths of time, and/or retraction of the published work.

Name: Junichiro Sageshima
Manuscript ID: K360-2025-000015R1
Manuscript Title: Packed Red Blood Cell and Whole Blood Perfusates during Ex-vivo Normothermic Perfusion for Assessment of High-Risk Donor Kidneys
Date of Completion: March 10, 2025
Disclosure Updated Date: March 10, 2025

ASN American Society of Nephrology | JASN Journal of the American Society of Nephrology | KIDNEY360 Accessing Our World From Every Angle | CJASN Clinical Journal of the American Society of Nephrology

ASN Journal Disclosure Form

As per ASN journal policy, I have disclosed any financial relationships or commitments I have held in the past 36 months as included below. I have listed my Current Employer below to indicate there is a relationship requiring disclosure. If no relationship exists, my Current Employer is not listed.

H. Yang reports the following:
Employer: University of California San Francisco

I understand that the information above will be published within the journal article, if accepted, and that failure to comply and/or to accurately and completely report the potential financial conflicts of interest could lead to the following: 1) Prior to publication, article rejection, or 2) Post-publication, sanctions ranging from, but not limited to, issuing a correction, reporting the inaccurate information to the authors' institution, banning authors from submitting work to ASN journals for varying lengths of time, and/or retraction of the published work.

Name: Heiko Yang
Manuscript ID: K360-2025-000015R1
Manuscript Title: Packed Red Blood Cell and Whole Blood Perfusates during Ex-vivo Normothermic Perfusion for Assessment of High-Risk Donor Kidneys
Date of Completion: April 10, 2025
Disclosure Updated Date: April 10, 2025
