# Supplementary material for: Packed Red Blood Cell and Whole Blood Perfusates during Ex Vivo Normothermic Perfusion for Assessment of High-Risk Donor Kidneys
Source: Kidney360. 2025 May 7;6(9):1573–85. doi: 10.34067/KID.0000000815 (PMC12503136; doi:10.34067/KID.0000000815)
Supplement: Supplementary file 2 [file kidney360-6-01573-s002.pdf]

Supplemental Material

Supplemental Figure 1. Metabolic pathway analysis of changes within WB/PRBC groups and temporal derangements comparing the two groups. A) Pre vs post WB, B) pre vs post PRBC, and C) WB vs PRBC over time. The size and color of the nodes represent pathway impact value and *P* value, respectively.

Supplemental Figure 2. Tissue lipid baseline differences comparing WB and PRBC. "x" represents a P <0.05 "xx" represents P <0.001

Supplemental Table 1. Tissue temporal metabolic differences among the WB group comparing pre and post perfusion (n=4).

Metabolites	Fold change (T3/T0)	P-value	Pathway
Glucose	9.8	<0.01	Sugar
Ascorbic acid	9.2	<0.01	Vitamin
Trehalose	7.6	0.03	Sugar
Ornithine	6.8	<0.01	Urea cycle
Histidine	6.3	<0.01	Amino acid
5-hydroxymethyl-2-furoic acid	4.9	<0.01	nematicide
2-hydroxyvaleric acid	4.3	0.03	Branched-chain amino acid metabolites (valine)
Indole-3-acetate	3.9	0.04	Tryptophan metabolism
Alanine	3.9	0.04	Amino acid
Tryptophan	3.3	<0.01	Amino acid
Isoleucine	2.9	<0.01	Amino acid
3,6-anhydro-D-galactose	2.9	0.01	Sugar
Proline	2.8	0.02	Amino acid
Serine	2.6	<0.01	Amino acid
Cytidine-5-monophosphate	2.5	0.02	Pyrimidine metabolism
Tagatose	2.4	0.04	Sugar
Threonine	2.3	<0.01	Amino acid
Xanthine	2.2	0.01	purine metabolism
Valine	2.2	<0.01	Amino acid
Phenylalanine	2.1	<0.01	Amino acid
Methionine	2.1	0.04	Amino acid
Fumaric acid	2.1	<0.01	TCA cycle
Malic acid	1.9	0.01	TCA cycle
Alpha-ketoglutarate	1.9	0.04	TCA cycle
Leucine	1.8	0.01	Amino acid
Lactic acid	1.5	0.02	Glycolysis/TCA
Hydroxylamine	1.4	0.03	Antioxidant
Benzoic acid	1.3	0.02	amino acid metabolism
Doconexent	0.6	<0.01	alpha-Linolenic acid metabolism
Putrescine	0.5	0.04	Fatty acid metabolism
Lactobionic acid	0.5	0.04	Sugar
Beta-gentiobiose	0.5	0.04	Sugar
Beta-alanine	0.5	0.02	amino acid metabolism
1-kestose	0.5	0.04	Sugar
Ribonic acid	0.4	<0.01	Sugar acid
Hypoxanthine	0.4	0.02	purine metabolism
Phosphoethanolamine	0.3	0.01	Starch and sucrose metabolism
Maltose	0.3	0.03	Sugar
Isomaltose	0.3	<0.01	Sugar
Galactinol	0.3	<0.01	Galactose Metabolism
Cysteine	0.3	0.01	Amino acid
UDP-N-acetylglucosamine	0.2	0.02	amino acid metabolism
Dehydroascorbic acid	0.2	0.02	Vitamin

Supplemental Table 2. Tissue temporal metabolic differences among the PRBC group comparing pre and post perfusion (n=4).

Metabolites	Fold change (T3/T0) PRBC	P-value	Pathway
Glucose	8.3	<0.01	Sugar
Ornithine	6.4	<0.01	Urea cycle
2-hydroxyvaleric acid	5.6	0.03	Branched-chain amino acid metabolites (valine)
Citric acid	5.6	<0.01	TCA cycle
Alanine	5.4	<0.01	Amino acid
Histidine	4.7	0.02	Amino acid
Inosine-5'-monophosphate	4.0	0.02	Purine metabolism
Threonic acid	4.0	0.04	ascorbate and aldarate metabolism
5-hydroxymethyl-2-furoic acid	3.7	<0.01	nematicide
3,6-anhydro-D-galactose	3.6	0.01	Galactose metabolism
Glucoheptulose	3.4	<0.01	Sugar
Fructose-1-phosphate	3.1	0.02	Glycolosis
Serine	3.0	<0.01	Amino acid
Proline	2.9	<0.01	Amino acid
Tryptophan	2.9	0.04	Amino acid
3-hydroxybutyric acid	2.5	<0.01	Fatty acid metabolism
Isoleucine	2.5	0.02	Amino acid
Threonine	2.4	<0.01	Amino acid
Phenylalanine	2.0	0.04	Amino acid
Leucine	1.9	0.03	Amino acid
Azelaic acid	1.3	0.01	Fatty acid metabolism
Nicotinamide	1.3	0.03	Vitamin
Phosphate	0.8	<0.01	Electrolyte
Beta-gentiobiose	0.5	0.04	Sugar
Pseudo-uridine	0.5	0.04	Pyrimidine metabolism
Ribonic acid	0.5	<0.01	Sugar acid
Xylose	0.5	<0.01	Sugar
1-kestose	0.4	0.03	Sugar
Galactinol	0.4	0.03	Galactose Metabolism
Dehydroascorbic acid	0.3	0.02	Vitamin
Isomaltose	0.3	0.02	Sugar
Isothreonic acid	0.3	0.04	ascorbate and aldarate metabolism
Maltose	0.3	0.04	Sugar
Palatinitol	0.3	0.01	Sugar

Supplemental Table 3. Tissue temporal lipid profile differences among the WB group comparing pre and post perfusion (n=4).

Lipids	Fold change (T3/T0 WB)	P-value	Lipid class
CE (18:2)	6.3	0.03	Steroids
CE (20:5)	3.4	<0.01	Steroids
TG (56:5)	3.4	<0.01	Glycerolipids
CE (18:1)	3.3	0.04	Steroids
TG (48:0)	3.3	0.01	Glycerolipids
TG (54:6)	3.3	0.01	Glycerolipids
TG (52:1)	3.2	0.04	Glycerolipids
CE (20:3)	3.1	0.04	Steroids
CE (20:4)	3.0	0.04	Steroids
TG (50:1)	2.9	0.01	Glycerolipids
TG (48:1)	2.7	0.01	Glycerolipids
TG (50:2)	2.6	0.01	Glycerolipids
TG (52:2)	2.6	0.03	Glycerolipids
TG (56:6)	2.6	<0.01	Glycerolipids
TG (46:0)	2.5	0.04	Glycerolipids
TG (50:3)	2.5	0.04	Glycerolipids
TG (52:3)	2.4	0.02	Glycerolipids
TG (50:4)	2.3	0.03	Glycerolipids
TG (51:1)	2.3	0.03	Glycerolipids
TG (54:4)	2.2	0.03	Glycerolipids
CE (18:3)	2.1	0.04	Steroids
TG (51:3)	2.1	0.03	Glycerolipids
TG (52:4)	2.1	0.02	Glycerolipids
TG (52:5)	2.1	0.02	Glycerolipids
TG (56:7)	2.1	<0.01	Glycerolipids
TG (56:8)	2.1	0.03	Glycerolipids
TG (51:2)	2.0	0.03	Glycerolipids
TG (49:0)	1.9	0.04	Glycerolipids
TG (58:9)	1.9	0.03	Glycerolipids
TG (50:5)	1.8	0.01	Glycerolipids
TG (50:5)	1.8	<0.01	Glycerolipids
TG (56:2)	1.8	0.04	Glycerolipids
TG (58:4)	1.8	0.01	Glycerolipids
Ceramide (d38:1)	1.7	0.01	Sphingolipids
PC (p-42:4) or PC (o-42:5)	1.7	0.03	Glycerophospholipids
PC (p-44:4) or PC (o-44:5)	1.7	<0.01	Glycerophospholipids
CE (22:6)	1.6	0.04	Steroids
TG (54:7)	1.5	0.03	Glycerolipids
Ceramide (d40:1)	1.4	0.01	Sphingolipids
Ceramide (d40:1)	1.4	<0.01	Sphingolipids
Ceramide (d42:2)	1.4	0.02	Sphingolipids
PC (p-40:3) or PC (o-40:4)	1.3	0.01	Glycerophospholipids
PE (p-36:1) or PE (o-36:2)	1.2	0.04	Glycerophospholipids
PC (34:0)	1.1	0.03	Glycerophospholipids
docosahexaenoic acid	0.6	0.01	Fatty acyls
LPC (16:0)	0.6	0.04	Glycerophospholipids
LPC (16:0)	0.5	0.02	Glycerophospholipids
LPC (20:4)	0.2	0.03	Glycerophospholipids

Supplemental Table 4. Minimal temporal differences in tissue lipid profile among PRBC perfused kidneys (n=4).

	Fold Change T3/T0 PRBC	P-value	Lipid class
Ceramide (d34:0)	1.9	0.02	Sphingolipids
DG (38:3)	1.4	0.04	Glycerolipids
Ceramide (d40:1)	1.1	0.04	Sphingolipids
PC (38:5)	0.8	0.01	Glycerophospholipids
PC (40:6)	0.8	0.03	Glycerophospholipids
DG (38:6)	0.6	0.04	Glycerolipids
PC (40:7)	0.6	<0.01	Glycerophospholipids
PC (40:8)	0.6	0.01	Glycerophospholipids
LPC (18:0)	0.5	0.01	Glycerophospholipids
Acylcarnitine C18:0	0.4	<0.01	Fatty acyls
LPC (16:0)	0.4	0.03	Glycerophospholipids
LPC (18:1)	0.3	0.03	Glycerophospholipids
LPC (18:2)	0.1	0.03	Glycerophospholipids

Supplemental Table 5. Differences in tissue lipid profile comparing WB (n=4) and PRBC (n=4) over time.

	Fold change (T3/T0)WB	Fold change (T3/T0)PRBC	P-value for interaction	Lipid Class
CE (18:2)	3.987	0.341	0.044	Steroids
TG (56:5)	2.985	1.206	0.048	Glycerolipids
TG (54:6)	2.679	0.889	0.017	Glycerolipids
CE (20:4)	2.550	0.449	0.048	Steroids
TG (56:6)	2.437	1.148	0.040	Glycerolipids
TG (48:0)	2.423	0.836	0.012	Glycerolipids
TG (52:1)	2.422	0.967	0.035	Glycerolipids
CE (20:5)	2.234	0.670	0.019	Steroids
TG (46:0)	2.158	0.697	0.029	Glycerolipids
TG (50:1)	2.107	0.741	0.009	Glycerolipids
CE (18:3)	1.973	0.611	0.038	Steroids
TG (50:2)	1.921	0.670	0.013	Glycerolipids
TG (48:1)	1.896	0.588	0.019	Glycerolipids
TG (51:1)	1.867	0.774	0.019	Glycerolipids
TG (52:2)	1.860	0.691	0.006	Glycerolipids
TG (52:3)	1.810	0.651	0.007	Glycerolipids
TG (49:0)	1.796	0.976	0.010	Glycerolipids
TG (44:1)	1.777	0.791	0.036	Glycerolipids
TG (54:2)	1.746	0.815	0.039	Glycerolipids
TG (46:1)	1.736	0.646	0.027	Glycerolipids
PC (p-42:4) or PC (o-42:5)	1.703	0.997	0.020	Glycerophospholipids
TG (46:2)	1.701	0.683	0.039	Glycerolipids
TG (52:4)	1.700	0.676	0.010	Glycerolipids
TG (53:4)	1.669	0.665	0.009	Glycerolipids
TG (50:3)	1.667	0.583	0.022	Glycerolipids
PC (p-44:4) or PC (o-44:5)	1.659	0.923	0.018	Glycerophospholipids
TG (54:5)	1.655	0.841	0.008	Glycerolipids
TG (54:3)	1.652	0.697	0.015	Glycerolipids
TG (49:1)	1.630	0.770	0.021	Glycerolipids
TG (54:4)	1.628	0.675	0.018	Glycerolipids
TG (48:2)	1.614	0.582	0.035	Glycerolipids
TG (52:5)	1.607	0.673	0.008	Glycerolipids
TG (50:4)	1.605	0.619	0.018	Glycerolipids
TG (58:4)	1.604	1.160	0.040	Glycerolipids
TG (51:2)	1.594	0.711	0.037	Glycerolipids
TG (51:3)	1.593	0.627	0.030	Glycerolipids
TG (53:3)	1.577	0.638	0.044	Glycerolipids
TG (50:5)	1.533	0.818	0.004	Glycerolipids
TG (48:3)	1.526	0.593	0.036	Glycerolipids
TG (50:5)	1.519	0.780	0.015	Glycerolipids
TG (49:2)	1.463	0.746	0.036	Glycerolipids
TG (54:7)	1.462	0.861	0.019	Glycerolipids
CE (22:6)	1.391	0.715	0.032	Steroids
PC (p-40:3) or PC (o-40:4)	1.303	0.899	0.018	Glycerophospholipids
PC (33:0)	1.169	0.903	0.007	Glycerophospholipids
PC (p-34:0) or PC (o-34:1)	1.108	0.918	0.039	Glycerophospholipids
PC (36:1)	1.102	0.863	0.024	Glycerophospholipids
PC (32:0)	1.060	0.921	0.030	Glycerophospholipids

PE (36:1)	1.030	0.864	0.010	Glycerophospholipids
PC (40:4)	1.029	0.755	0.005	Glycerophospholipids
PC (31:0)	1.021	0.861	0.043	Glycerophospholipids
PE (p-38:4) or PE (o-38:5)	0.957	0.829	0.045	Glycerophospholipids
LPC (18:1)	0.398	0.244	0.003	Glycerophospholipids

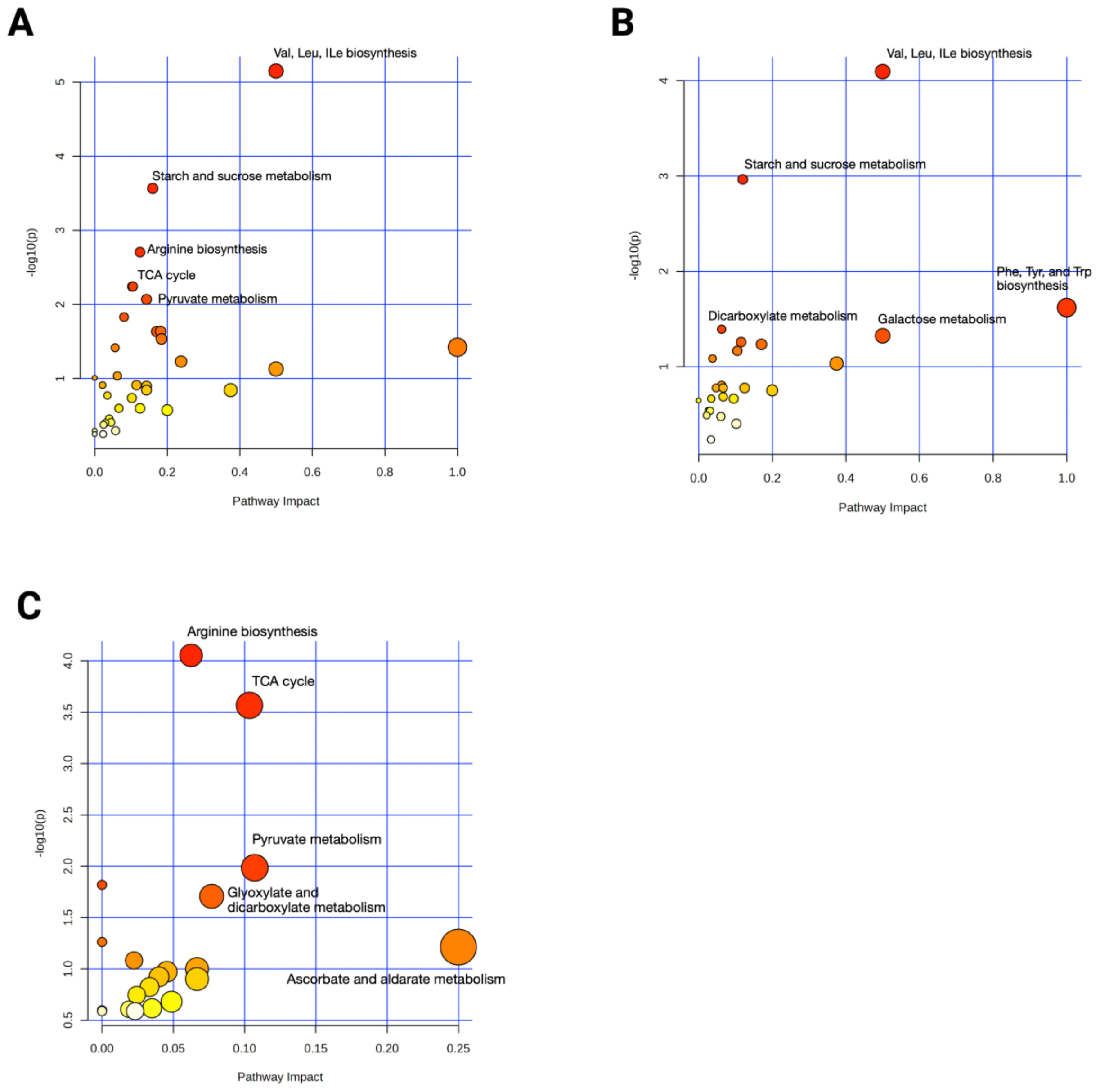
A
Val, Leu, ILe biosynthesis
Starch and sucrose metabolism
Arginine biosynthesis
TCA cycle
Pyruvate metabolism
-log10(p)
Pathway Impact
B
Val, Leu, ILe biosynthesis
Starch and sucrose metabolism
Phe, Tyr, and Trp biosynthesis
Dicarboxylate metabolism
Galactose metabolism
-log10(p)
Pathway Impact
C
Arginine biosynthesis
TCA cycle
Pyruvate metabolism
Glyoxylate and dicarboxylate metabolism
Ascorbate and aldarate metabolism
-log10(p)
Pathway Impact

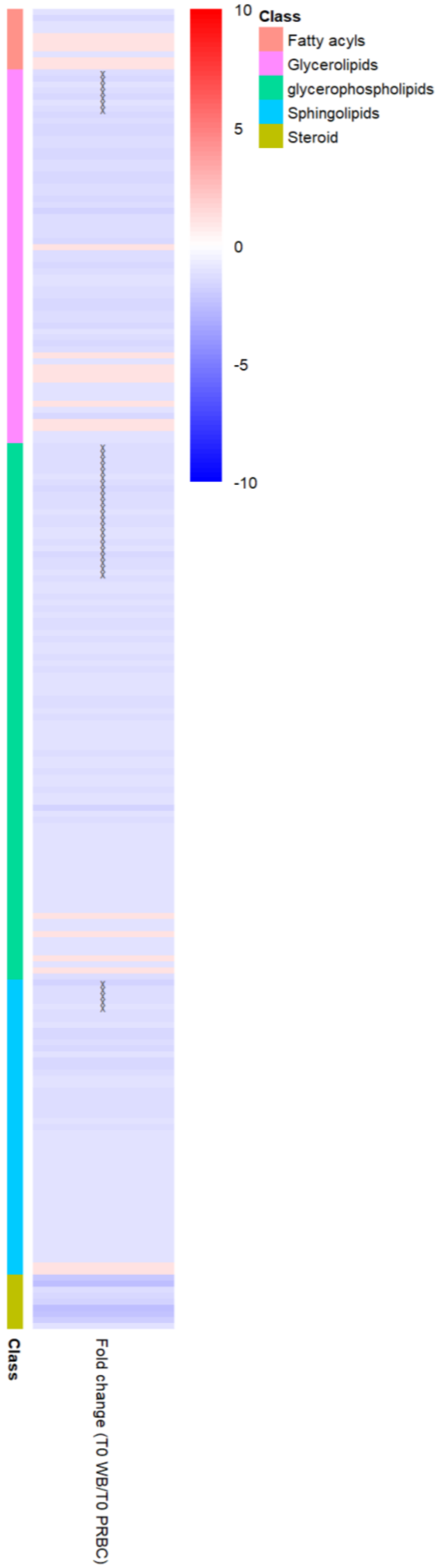

10
5
0
-5
-10
Class
Fatty acyls
Glycerolipids
glycerophospholipids
Sphingolipids
Steroid
Class
Fold change (T0 WB/T0 PRBC)
